# Genomic, expressional, protein-protein interactional analysis of Trihelix transcription factor genes in *Setaria italia* and inference of their evolutionary trajectory

**DOI:** 10.1186/s12864-018-5051-9

**Published:** 2018-09-12

**Authors:** Zhenyi Wang, Kanglu Zhao, Yuxin Pan, Jinpeng Wang, Xiaoming Song, Weina Ge, Min Yuan, Tianyu Lei, Li Wang, Lan Zhang, Yuxian Li, Tao Liu, Wei Chen, Wenjing Meng, Changkai Sun, Xiaobo Cui, Yun Bai, Xiyin Wang

**Affiliations:** 1 College of Life Sciences, North China University of Science and Technology, Caofeidian Dist, Tangshan, 063210 Hebei China; 2 Center for Genomics and Computational Biology, North China University of Science and Technology, Caofeidian Dist, Tangshan, 063210 Hebei China; 3 College of Science, North China University of Science and Technology, Caofeidian Dist, Tangshan, 063210 Hebei China

**Keywords:** Foxtail millet, Trihelix, Transcription factor, Grass, Evolution, Selection

## Abstract

**Background:**

Trihelix transcription factors (TTF) play important roles in plant growth and response to adversity stress. Until now, genome-wide identification and analysis of this gene family in foxtail millet has not been available. Here, we identified TTF genes in the foxtail millet and its grass relatives, and characterized their functional domains.

**Results:**

As to sequence divergence, TTF genes were previously divided into five subfamilies, I-V. We found that Trihelix family members in foxtail millet and other grasses mostly preserved their ancestral chromosomal locations during millions of years’ evolution. Six amino acid sites of the SIP1 subfamily possibly were likely subjected to significant positive selection. Highest expression level was observed in the spica, with the SIP1 subfamily having highest expression level. As to the origination and expansion of the gene family, notably we showed that a subgroup of subfamily IV was the oldest, and therefore was separated to define a new subfamily O. Overtime, starting from the subfamily O, certain genes evolved to form subfamilies III and I, and later from subfamily I to develop subfamilies II and V. The oldest gene, Si1g016284, has the most structural changes, and a high expression in different tissues. What’s more interesting is that it may have bridge the interaction with different proteins.

**Conclusions:**

By performing phylogenetic analysis using non-plant species, notably we showed that a subgroup of subfamily IV was the oldest, and therefore was separated to define a new subfamily O. Starting from the subfamily O, certain genes evolved to form other subfamilies. Our work will contribute to understanding the structural and functional innovation of Trihelix transcription factor, and the evolutionary trajectory.

**Electronic supplementary material:**

The online version of this article (10.1186/s12864-018-5051-9) contains supplementary material, which is available to authorized users.

## Background

Transcription factor is a type of DNA binding protein, and interacts with cis element of promoter regions of target genes, regulating the expression of them. At present, more than 60 transcription factor families have been found in plants [1]. Trihelix transcription factor is among the earliest transcription factor families discovered in plants [1].

Trihelix transcription factors (TTF) feature a conservative domain containing three series of alpha helix structure [2, 3]. TTFs were reported to play multiple regulatory roles in plant growth, development process, and response to adversity stress [4–7]. According to the changes in their alpha helix domain [8], they were previously divided into five subfamilies, respectively referring as I(or SH4), II(or GT-1), III(or GTγ), IV(or SIP1), and V(or GT-2). Each subfamily was named as to their respective first member found. Pea (*Pisumsativum l*.) GT-1 factor is the earliest identified TTF, which specifically combined with GT elements of light-induced gene *rbcS–3A*’s promoter [4]. In tobacco (*Nicotiana tabacum*) [6], *Arabidopsis* (*Arabidopsis thaliana*) [7], and rice (*Oryza sativa*) [5], homologous GT-1 genes were cloned. GT-2 was the first GT-factor isolated, containing two separate Trihelix domains [9, 10], each involved in DNA binding. *Arabidopsis*’s *ETAL LOSS (PTL)* gene belongs to the GT-2 family, and can regulate the growth of petals and sepals. It was also found to regulate flower organ formation of shape [11–13]. Rice *SHATTERING1 (SHA1)* gene, encoding a SH4 type of transcription factor, is the only identified member found in the SH4 subfamily, playing an important role in cell differentiation activation. A mutant *SHA1* gene was found to cause the disappearance of the seed holding in rice [14]. GTγ subfamily has four members identified in rice, *Os*GTγ-1、*Os*GTγ-2、*Os*GTγ-3, and *Os*GTγ-4, which were related to cold, drought, and salt stress response [15]. Certain SIP1 genes have been identified in the tobacco and *Arabidopsis*, related to the development of plant embryo, leaf development, and cell proliferation [16–18]. Recently, expression profiles of Trihelix genes were available in tomato [19] and *Populus trichocarpa*, under biotic and abiotic stresses in the latter [20]. A new gene *BnSIP1* was discovered in *Brassica napus* [21] mediating abiotic stress tolerance and ABA signaling.

Foxtail millet (*Setaria italica*) is one important arid and semi-arid crop, being a staple diet for people in some regions in China, India, and other Asian countries. Owing to its economic importance, its genome was sequenced [22, 23], together with further sequencing efforts [24–27], providing a rich genomic and genetic resources for biological research and breeding practice [28]. These precious efforts and accumulating resources empower researches in the Setaria community. Recently, tens of researches were performed to understand key functional gene families of the crop [29–43]. These researches described certain important transcription factor genes and gene families, such as Dof genes, encoding a class of transcription factors involved in numerous physiological and biochemical reactions affecting growth and development [43], *TRANSPARENT TESTA GLABRA 1* genes, encoding a WD40 repeat transcription factor with multiple roles in plant growth and development, particularly in seed metabolite production [41], lipid transfer protein genes (LTPs), encoding a class of cysteine-rich soluble proteins having small molecular weights [38], MYB genes [44], APETALA2/ethylene-responsive element binding factor (AP2/ERF) genes [45], NAC genes [46] and so on .

Here, we identified TTF genes in foxtail millet, and characterized their molecular characteristic, genome distribution, and possible biological function. Moreover, by performing an evolutionary genomics analysis in selected plants, moss, green algaes, and yeast, we explored the evolution and origin of the TTF genes and inferred their possible evolutionary trajectories about their origin and divergence.

## Methods

### Data collection

Genome data of foxtail millet, rice, and sorghum were downloaded from JGI database (http://genome.jgi.doe.gov/). To identify putative Trihelix family members, the Hidden Markov Model (HMM) profiles of Trihelix (PF13837) were retained from the Pfam database (http://pfam.xfam.org/) and were used to identify the putative Trihelix proteins with the best domain e-value cutoffs of < 1 × 10^− 4^. The rice Trihelix sequences [1] were used as the query to perform a BLASTP search in these species [47], with a cutoff e-value of< 10^− 10^. By using SMART program [48] (http://smart.embl-heidelberg.de/) and the National Center for Biotechnology Information (NCBI) database (http://www.ncbi.nlm.nih.gov/), we detected the candidate protein by characterizing the typical Trihelix feature structure domain. We checked the ExPASy database (http://www.expasy.org/) to retrieve information as molecular weight, isoelectric point of TTF proteins [49]. Based on the above method and TFDB 4.0 database (http://planttfdb.cbi.pku.edu.cn/) [50], we obtained TTF homologs from other species: *Ae. tauschii*, *T. urartu*, barley, *Brachypodium*, maize, *Saccharomyces cerevisiae, Chlamydomonas reinhardtii, Coccomyxa subellipsoidea, Volvox carteri, Physcomitrella patens,* and *Selaginella moellendorffii*.

### Gene structure analysis

According to the downloaded gff3 annotation file, the required data is extracted and the format is modified by the home-made Perl program. By using GSDS 2.0 (http://gsds.cbi.pku.edu.cn/), we analyzed genetic structure of TTF genes [51].

### Motif identification

By using protein conservative motif online search program MEME 4.11.3 (http://meme-suite.org/tools/meme), we analyzed conservative motif of TTF gene family, and set the relevant parameters of motif repeat number to be “any”, motif length to be 6 ~ 200 aa, and motif prediction number to be 25 [52, 53]. By using WebLogo 3.6.0 (http://weblogo.threeplusone.com/), we characterized conservative region in amino acid sequence [54].

### Gene localization and divergence

We used BioPerl program to estimate synonymous nucleotide substitution per synonymous site (*Ks*), and then drawing the circle diagram through the home-made Python program. All millet Trihelix genes are noted in the chromosome, genome evolution homologous duplicate events are connected by color lines with *Ks*. *Ks*: 0–0.35 black; 0.35–0.45 green; 0.45–0.65 red; 0.65–2 blue [55].

### Multiple sequence alignment and evolutionary tree construction

Multiple sequence alignment of millet, rice, sorghum, *Ae. tauschii, T.urartu,* barley, *Brachypodium* and maize TTF gene family were performed by using Clustal X version 2.0 [56]. According to the sequence alignment, phylogenetic tree of TTF genes were built by PHYLIP 3.695 program with the Neighbor-joining method (http://evolution.genetics.washington.edu/phylip.html), and the Bootstrap value 1000 was adopted.

### Selection pressure analysis

Using PAML 4.8 Codeml program (http://abacus.gene.ucl.ac.uk/software/paml.html), we tested whether the sequences to bear the positive selection with four comparison models of M1a, M2a, M7, and M8 [57].

### Orthologs in foxtail millet, rice and sorghum

Using OrthoMCL program (http://orthomcl.org/orthomcl/) [58], we analyzed chromosome segments duplication between foxtail millet, rice, and sorghum Trihelix genes, with the default settings, which initially required an all-against-all BLASTP, and then the relationships between the genes were deduced by the MCL clustering algorithm. The result is graphic by Circos software (http://circos.ca/) [59].

### Expression analysis

Transcriptome and RNA - seq data was downloaded from the foxtail millet database (http://foxtailmillet.genomics.org.cn/page/species/index.jsp), and TTF expression data extracted by using home-made Perl program. The foxtail millet TTF genes expression cluster from each tissue was analyzed using Cluster 3.0 software (http://bonsai.hgc.jp/~mdehoon/software/cluster/software.htm), and the RPKM values were log2 transformed. The heat map of hierarchical clustering was visualized with TreeView1.1.3.

### Protein interaction network

We used STRING 10.5 database (http://string.embl.de/) [60] to analyze millet TTF interaction with other foxtail millet proteins. We set the minimum required interaction score to be high confidence (0.700), and max number of interactors to be 5.

## Results

### Identification and genomic distribution

We identified 27 TTF genes in the foxtail millet genome database (Additional file 1: Table S1). The shortest sequence has 212 amino acid residues, while the longest one has 878 amino acid residues. The estimated protein molecular weights fall in a range 23,453.7~ 96,360.6, and the isoelectric points in a range 4.9184~ 11.2729.

The predicted 27 millet TTF genes have 36 transcripts (Additional file 2: Figure S1). Twenty-one genes (21 or 77.8%) were found to have a single transcript, while 6 of them have multiple transcripts, with Si7g009787 having the most (5). They have considerably divergent genic structures, with 1–17 exons. For example, 12 genes, e.g., Si6g014062, Si9g036682, have a single exon, while the gene Si1g016284 has 17 exons and its gene structure is broken into short pieces by inserted introns.

**Fig. 1:**
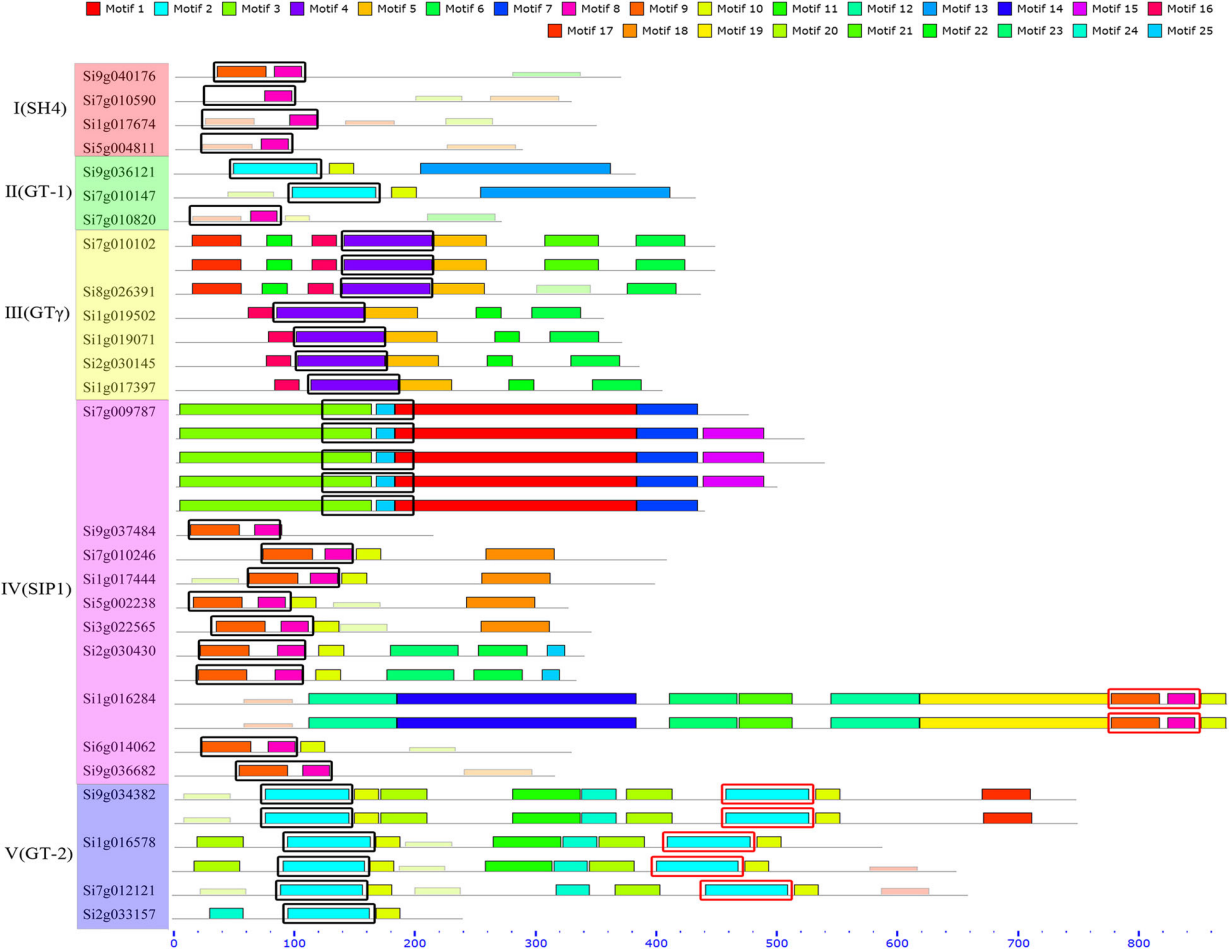
Millet Trihelix transcription factor family conservative motif analysis. Dark color pieces were generated by MEME software, light color pieces show possible Motif (using a motif scanning algorithm). The areas enclosed by boxes are a conserved domain, black indicates the N-terminal, and red indicates the C-terminal

We characterized the motif in the TTFs and found that they are diverse in motif composition, supporting previous finding of divergent evolution with characterization of exons and introns. Identified motifs often contain > = 15 amino acid residues even 200 amino acid residues. Some motifs, such as Motif 8, are conserved in different subfamilies (Fig. 1), while other motifs shared by subfamilies are much variable (Additional file 3: Table S2).

In foxtail millet, TTF genes in each subfamily have similar motif (Fig. 1). All six subfamily III genes contain Motif 4. The subfamilies I and IV feature the containing of Motif 8 and 9 while the subfamily II and the subfamily V features Motif 2.

TTF genes contain a conservative structure domain in the N terminal (except Si1g016284) (Fig. 1), while GT-2 contains the domain structure in the C terminal and 2 repeatitive and conservative structure domain. The GT-1 and GT-2 subfamilies are much more similar than to other subfamilies.

With the exception of Si1g016284, the other genes contain a conservative domain near the N-terminal, in which 1/5 of the amino acid residues are quite conservative, with Trp (W) - 1, Trp (W) - 64 and Cys(C) - 100 being highly conserved (Additional file 4: Figure S2).

**Fig. 2:**
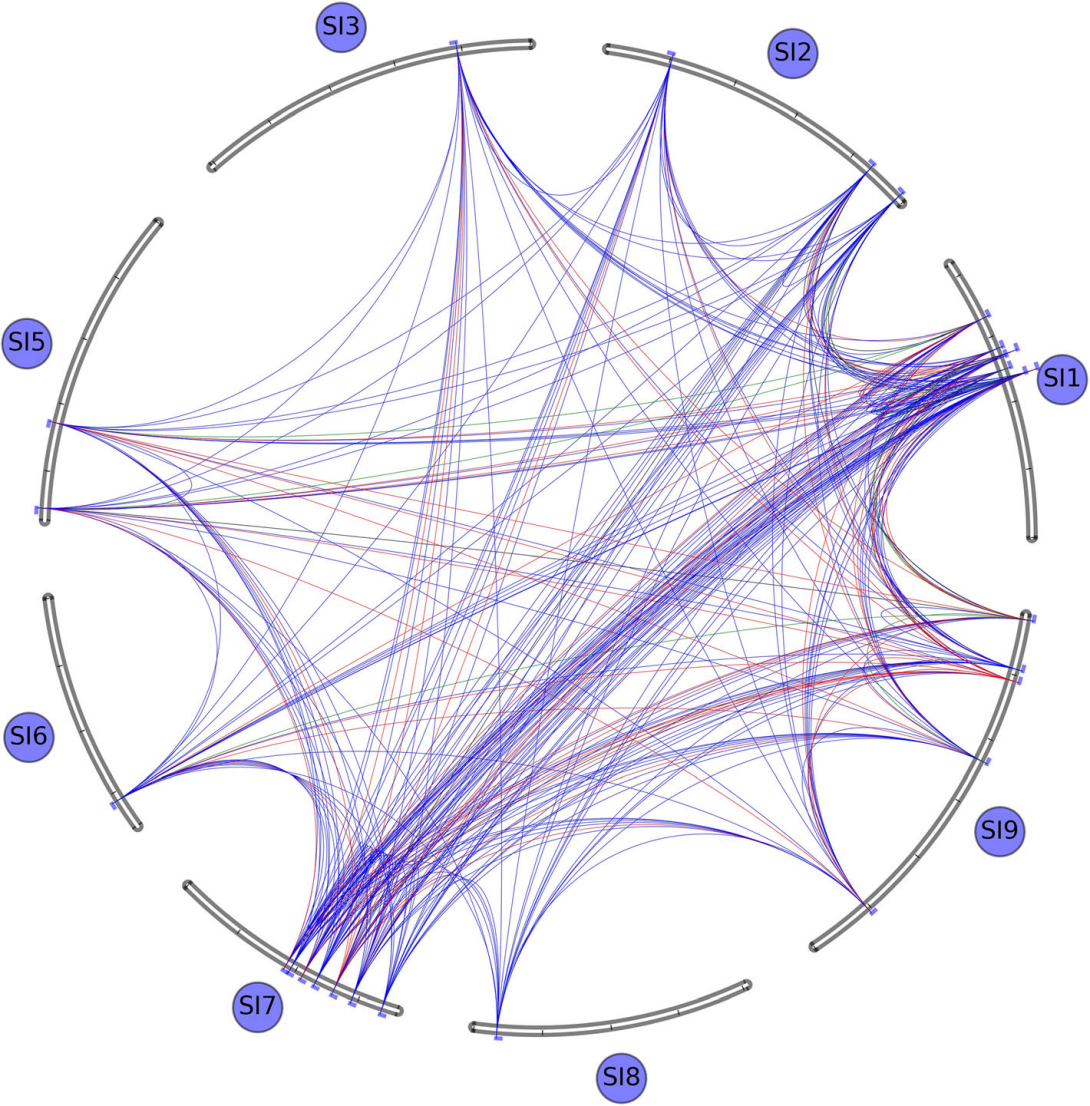
Millet Trihelix transcription factor family duplication analysis in the chromosome. All millet Trihelix genes are noted in the chromosome, genome evolution homologous duplicate events are connected by color lines with *Ks*. *Ks*: 0–0.35 black; 0.35–0.45 green; 0.45–0.65 red; 0.65–2 blue

According to gene localization in the foxtail millet genome, we found that, TTF genes are distributed in 8 foxtail millet chromosomes but chromosome 4, with chromosome 1 and 7 having 7 genes, chromosome 3, 6 and 8 having only 1 gene, and the others having 2–5 genes (Fig. 2). On chromosome 1 and 7, they form small clusters distributed in their middle and ending parts. Besides, there are 11 genes with *Ks* < 0.35, including a subfamily I gene (Si5g004811), 2 subfamily II genes (Si7g010147, Si9g036121), 4 subfamily III genes (Si1g017397, Si1g019071, Si1g019502, Si2g030145), 4 subfamily V genes (Si2g033157, Si1g016578, Si9g034382, Si7g012121), showing possible gene divergence after foxtail millet’s split from sorghum [55].

### Evolutionary establishment of the family

**Fig. 3:**
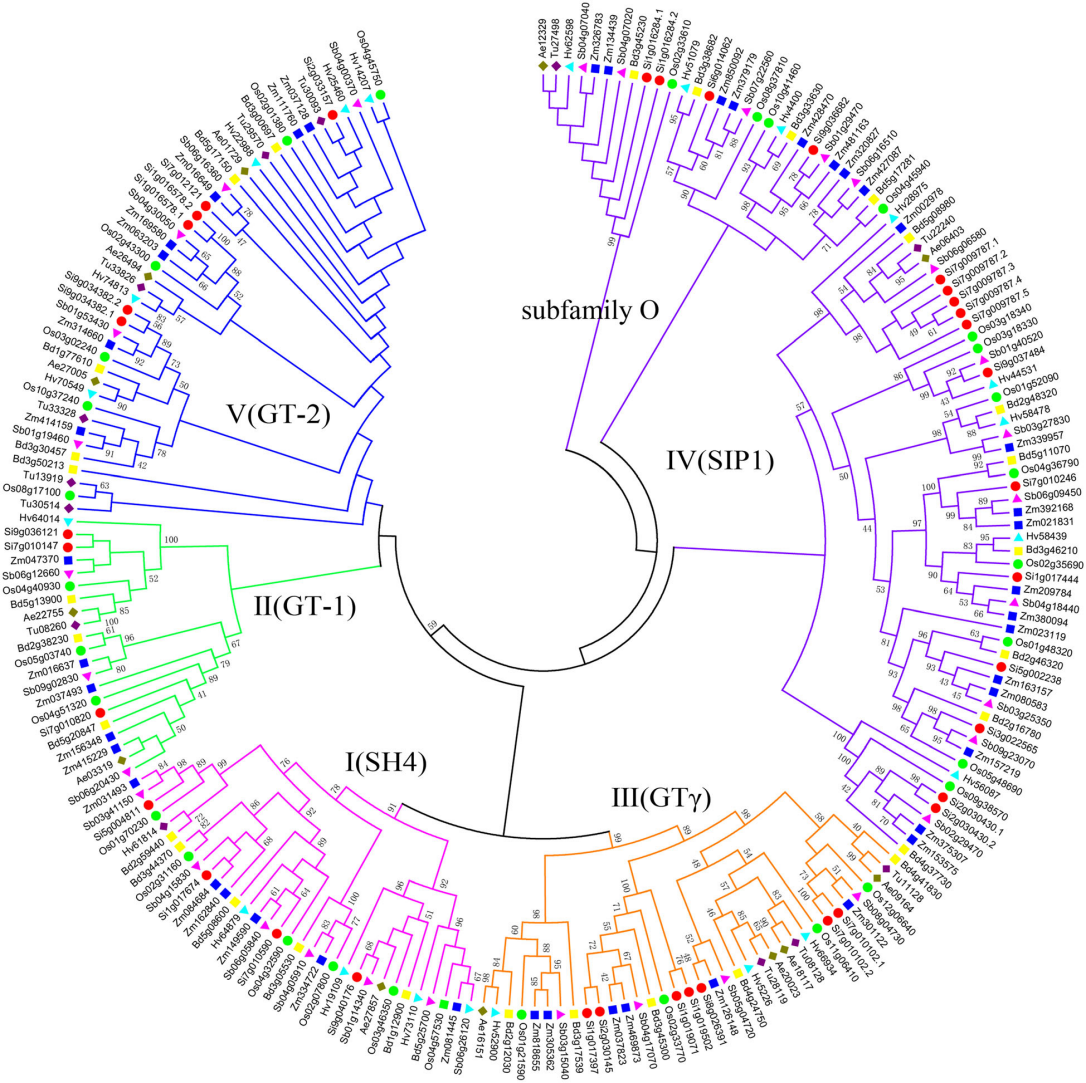
Reconstructed phylogenetic tree of grass TTF genes. Here, gene IDs show their respective origin: Os for rice, Si for *Setaria italia*, Sb for sorghum, Ae for *Aegilops tauschii*, Tu for *Triticum urartu*, Hv for barley, Bd for *Brachypodium*, and Zm for maize. We used shapes and colors to distinguish different species, with red circles, green circles, blue triangles, light pink triangles, blue squares, yellow squares, brown diamonds, deep purple diamonds to represent the TTF genes in *Setaria italia*, rice, barley, sorghum, maize, *Brachypodium*, *Aegilops tauschii*, and *Triticum urartu*, respectively. The number on the branches is support value by bootstraping

To understand the evolution of the gene family, we involved their homologous genes from its grass relatives, rice (*Oryza sativa*), sorghum (*Sorghum bicolor*), *Aegilops tauschii*, *Triticum urartu*, barley (*Hordeum vulgare*), *Brachypodium* (*Brachypodium distachyon*), and maize (*Zea mays*). Firstly, by using PHYLIP, we reconstructed the phylogenetic tree of TTF genes (Fig. 3). These grasses share genes from each subfamily, excepting *T. urartu*, in which none subfamily I gene was found in the present genome sequence. The subfamily IV has the most members in all species.

**Fig. 4:**
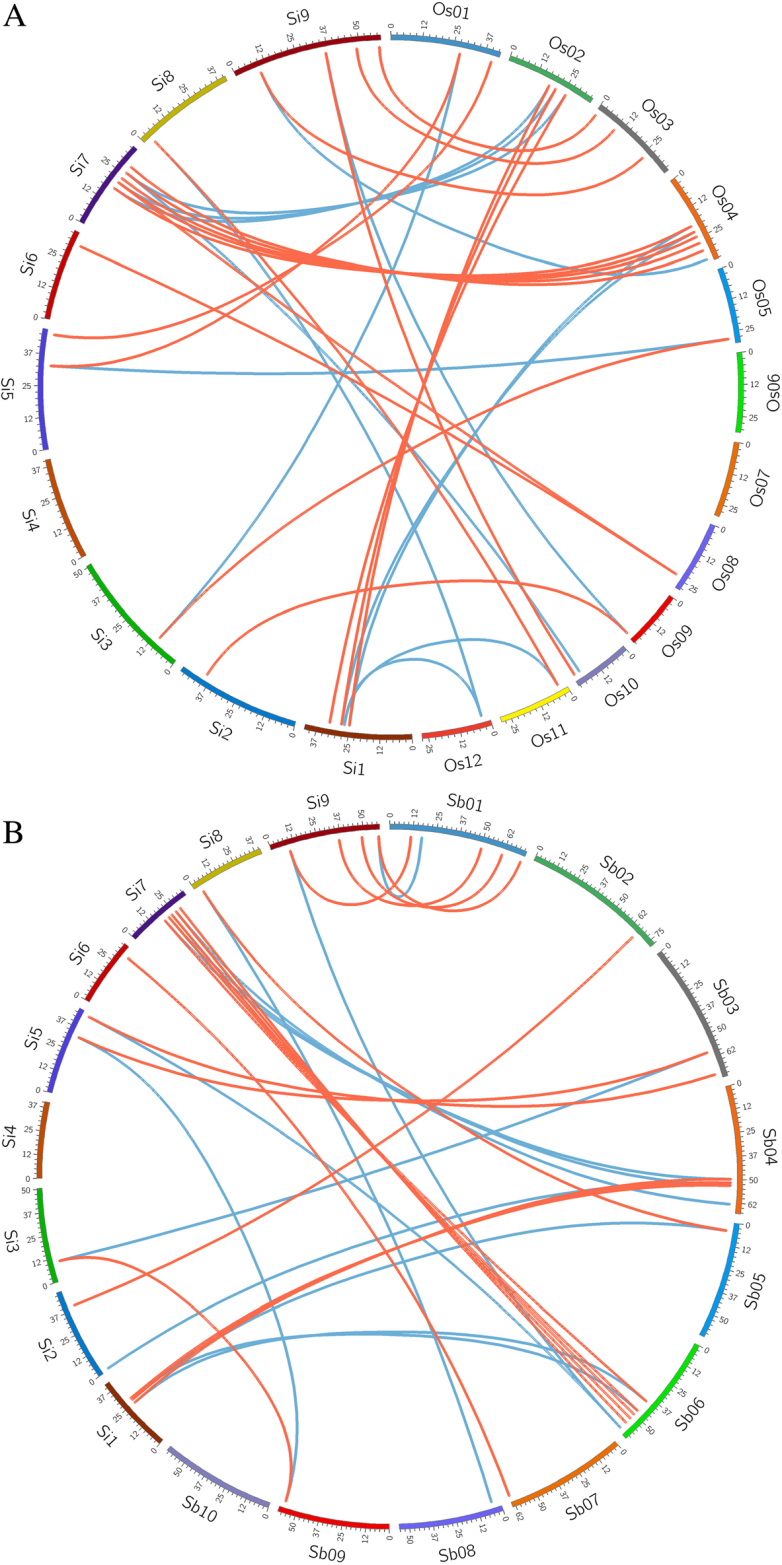
Colinearity analysis of TTF genes between foxtail millet, rice, and sorghum. Chromosomes from any two grasses form a circle, and a pair of collinear TTF genes are linked with a curvy line in red and blue, showing orthologous pairs or paralogous ones. Millet chromosomes: Si1 ~ Si9; Rice chromosomes: Os01~Os12; Sorghum chromosomes: Sb01~Sb10. **a**: Millet-Rice. **b**: Millet-Sorghum

Through the OrthoMCL program, we identified 32 TTF homologous gene pairs in millet and rice, including 19 orthologous gene pairs and 13 non-orthologous ones (Fig. 4a). Millet and sorghum share 31 colinear genes, including 18 orthologs in colinearity (Fig. 4b). The orthologous pairs are those homologs at the anticipated genomic locations from two genomes and often the best 1–1 match. For a non-orthologous pair, a millet gene may have another non-best hit in the other grass, many quite likely related to the grass-common tetraploidization occurring ~ 100 million years ago. Often these non-orthologous pair could be called as outparalogous pair. A total of 9 genes (Si1g017444, Si1g017674, Si3g022565, Si5g002238, Si7g010246, Si7g010590, Si7g012121, Si8g026391, Si9g040176) are conservative in chromosomal locations in all 3 genomes, showing their existence in grass common ancestor.

**Fig. 5:**
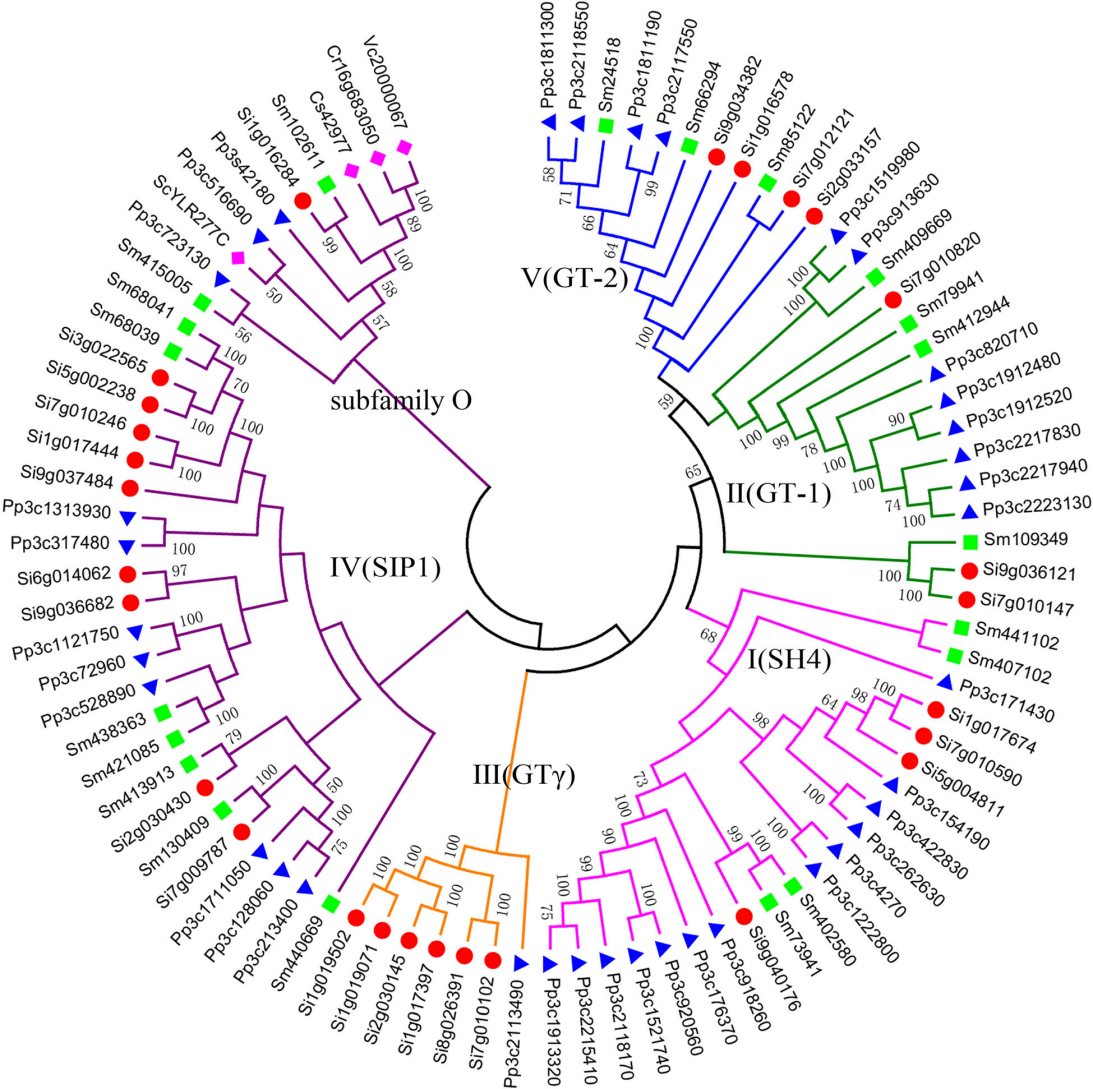
Reconstructed phylogenetic tree of TTF genes in involved species. Here, gene IDs show their respective origin: Si for *Setaria italia*, Pp for moss, Sm for fern, Sc for yeast, Cr for *Chlamydomonas reinhardtii*, Cs for *Coccomyxa subellipsoidea*, and Vc for *Volvox carteri*. We used shapes and colors to distinguish different species, with red circles, blue triangles, and green squares to represent TTF genes in *Setaria italia*, moss, fern, respectively and pink diamonds to represent TTF genes in yeast, *Chlamydomonas reinhardtii*, *Coccomyxa subellipsoidea*, and *Volvox carteri*. The number on the branches is support value by bootstraping

To find their a deeper history of the family, we reconstructed a phylogenetic tree involving homologs from representative organisms from different domains, including *Saccharomyces cerevisiae* (yeast), *Chlamydomonas reinhardtii* (green algae), *Coccomyxa subellipsoidea* (green algae), *Volvox carteri* (green algae), *Physcomitrella patens* (moss), *Selaginella moellendorffii* (fern), and foxtail millet (Fig. 5). Notably, the involved genes from these organisms can also be classified into 5 previously defined subfamilies (I~V) in grasses. There is only one TTF-like gene found in the algae and yeast (too old to form a Trihelix characteristic domain), while *Physcomitrella patens* and *Selaginella moellendorffii* have 37 and 20 TTF genes, respectively. A close check of the subfamily IV helped identify a certain group of genes, involving copies from the yeast and algae genes, and plant genes, therefore had existed before the divergence of major life domains. Therefore, we separated them from other subfamily IV genes, to define them as an extra group, or subfamily O. That is, with homologs from all species, we divided TTF genes into six subfamilies.

Genes forming subfamily IV were much diverged, involving the oldest lineages. Thus, we chose the subfamily IV to perform a natural selection analysis. By using the PAML Codeml program to perform likelihood ratio test, we estimated selective pressure on each lineage of the constructed tree. We found that 6 amino acid sites were likely subjected to significant positive selection (Table 1).

**Table 1:** Natural selection pressure analysis

	Pr (w > 1)	post mean for w
1 S	0.975*	2.942
28 D	0.585	1.851
81 L	0.791	2.434
93 Q	0.894	2.718
102 S	0.874	2.663
103 K	0.553	1.782
104 P	0.816	2.497
105 L	0.949	2.869
106 A	0.962*	2.903
107 T	0.974*	2.937
108 A	0.876	2.669
109 E	0.878	2.673
115 E	0.549	1.764
130 R	0.782	2.409
138 M	0.961*	2.903
141 S	0.888	2.701
142 V	0.948	2.865
144 V	0.754	2.328
165 A	0.971*	2.931
172 V	0.960*	2.900
183 T	0.948	2.865
186 A	0.866	2.640

Positively selected sites (*: *P* > 95%; **: *P* > 99%)

### Expression profile in the different organs

**Fig. 6:**
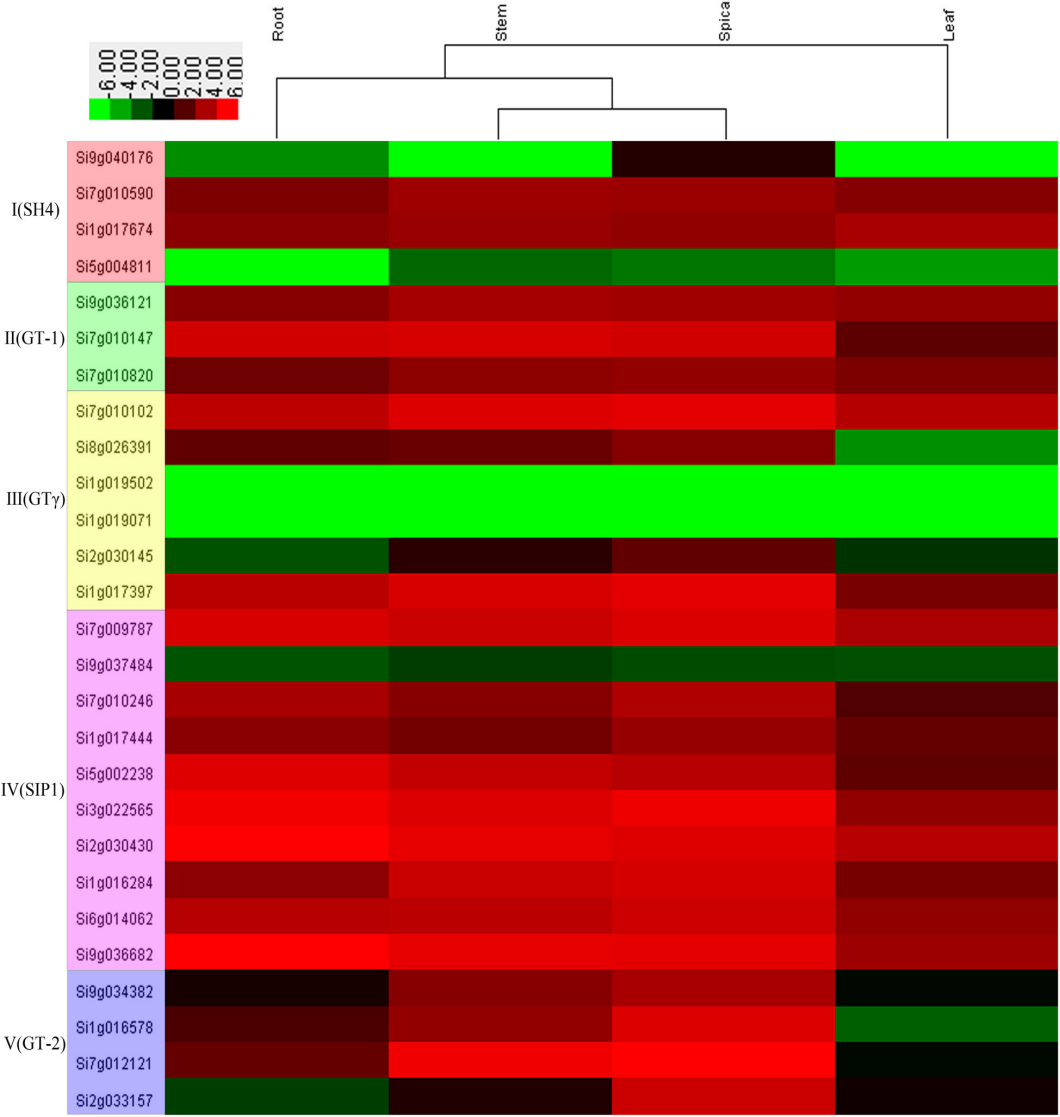
Expression of millet Trihelix transcription factor genes in the different organs

We adopted heat map to display expression profile of millet TTF genes from different tissues, involving root, stem, spica and leaf (Fig. 6, Additional file 5: Table S3). Here, we define the standard for high expression gene is more than the average expression of all genes(the average RPKM value is 15.7). There were 9 genes (33.3%), 11 genes (40.7%), 14 genes (51.9%) and 3 genes (11.1%) with high expression in root, stem, spica and leaf, respectively. In all organs, genes in spica had the highest expression level. Subfamily IV had the highest expression level in all subfamilies. Si1g019071 and Si1g019502 were not observed to be expressed in any tissues, Si5g004811 not in the root, and Si9g040176 not in the stem and leaf.

In addition, expression has been down-regulated in many structurally variable genes. Si9g040176 had more copies of Motif 9 than others in subfamily I, possibly subjected a recent motif addition and is down-regulated. In subfamily III Si8g026391 had fewer copies of Motif 21 than the genes Si7g010102, and Si9g037484 had the simplest structure in the subfamily IV indicating a motif deletion, and they are also down-regulated. In the subfamily V, compared to other genes, Si2g033157 lost the domain in the C terminal region, and it is down-regulated. In contrast, though Si7g009787, having the most transcripts, and Si1g016284, being the oldest gene in the family, are each variable in structure, they had higher expression, showing possible functional benefit of plants due to their variable structure.

### Protein-protein interaction

**Fig. 7:**
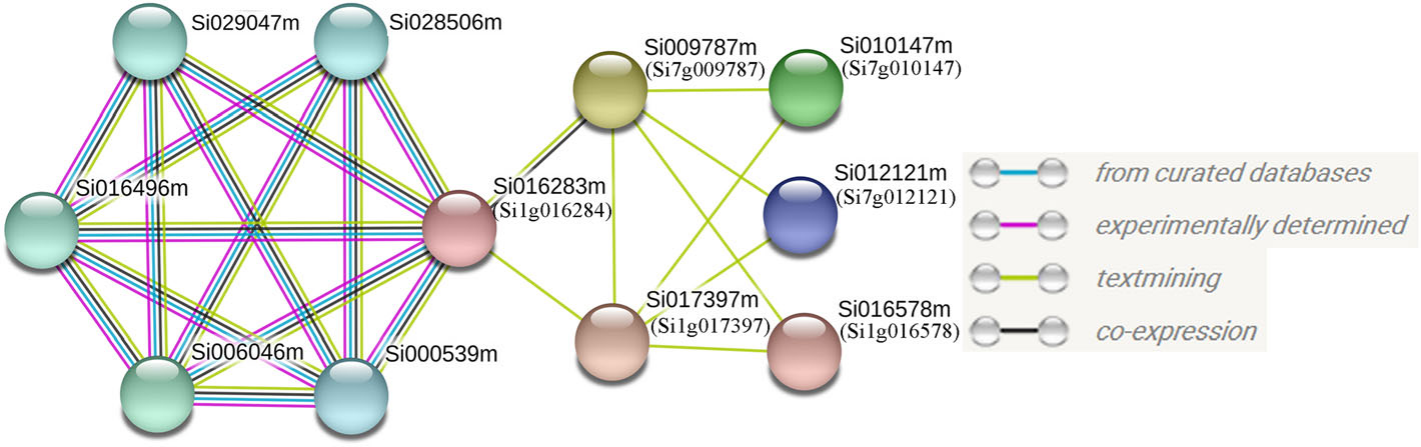
Interaction network diagram of Trihelix transcription factor and other proteins in foxtail millet

Protein interaction analysis shows that six of the foxtail millet Trihelix transcription factor families have interaction relationship (Fig. 7). Among them, Si1g016284 belongs to subfamily O, Si7g009787 belongs to subfamily IV, Si1g017397 belongs to subfamily III, Si7g010147 belongs to subfamily II, Si7g012121 and Si1g016578 belong to subfamily V. The protein-protein interaction information is from curated databases and experimentally determined. In addition, we also introduced textmining and co-expression to enrich the interaction information. We found that Si1g016284 from subfamily O has the most interaction with other proteins (7). It also has interaction with Si7g009787 and Si1g017397, which have five interacting proteins respectively. The genes, Si7g010147, Si7g012121 and Si1g016578 have two interacting proteins respectively.

The oldest TTF gene, Si1g016284, played a significant role in the interaction. It seems to serve as a bridge connecting the Trihelix family and other millet proteins, and is co-expressed with many proteins (6), suggesting that these proteins function synergistically. Structurally, Si1g016284 has two extra domains, Lactamase_B and RMMBL, in addition to the characteristic of the Trihelix family. The five non-Trihelix proteins interacting with it have variable domains, such as Lactamase_B, RMMBL, Beta_Casp, CPSF100_C, WD40, ZnF_C3H1, YTH, and/or HAT, showing a multiple-facet nature of Silg016284.

## Discussion

As a multi-functional transcription factor family, TTFs were the first ones identified in plants [1]. Here, starting from research in foxtail millet and extending into other organisms, we explored their functional changes, expressional features, genomic duplication and phylogenetics. Eventually, we identified an oldest subfamily, referred as O, in the constructed phylogenetic tree. Interestingly, the single foxtail millet gene Si1g016284 in subfamily O is the one having the most exons (Additional file 2: Figure S1). It has a single ortholog in yeast or any algae species, and two orthologs in fern and three orthologs in moss. Actually, this seems to be weird in that we would have expected that it might be the most conservative one to have highest similarity with the homologs from far diverged life domains. This shows that, though broken into 17 segments, the gene might have not been pseudogenized but rather likely functional.

Starting from the subfamily O, primitive TTF genes continued to expand in the plant domain. As to the reconstructed tree topology, we found that certain genes evolved to form subfamilies III and I, and later from subfamily I to develop subfamilies II and V (Fig. 5).

In each subfamily, there is evidence that genome duplications contributed to accumulate more copies. For example, in foxtail millet, a group of genes in subfamily IV appeared after its divergence from other grasses (Fig. 3), and moss has the most TTF genes with new copies seemingly having been continuously produced (Fig. 5).

The primitive TTF gene, Si1g016284, has conserved domain in its C terminal region, as genes forming subfamily O from different life domains. Contrastively, the conserved domains were found in N terminal or both terminals in the other foxtail millet genes (Fig. 1).

Besides, subfamily GTγ were not found in Lycophta and *S. Moellendorffii* (Fig. 5), consistent to previous report [61]. This shows that though as an old subfamily, they may have been pseudogenized or removed from certain plants.

## Conclusions

TTF genes were previously divided into five subfamilies, I-V. By performing phylogenetic analysis using non-plant species, notably we showed that a subgroup of subfamily IV was the oldest, and therefore was separated to define a new subfamily O. Starting from the subfamily O, certain genes evolved to form other subfamilies. The oldest gene, Si1g016284, has the most structural changes, and a high expression in different tissues. What’s more interesting is that it may have bridge the interaction with different proteins. Our work will contribute to understanding the structural and functional innovation of Trihelix transcription factor, and the evolutionary trajectory.

## Additional files

Additional file 1: **Table S1**. Basic information of foxtail millet Trihelix transcription factor genes. (DOCX 15 kb)

Additional file 2: **Figure S1**. Millet Trihelix family genetic structure analysis. (JPG 1489 kb)

Additional file 3: **Table S2**. Conservative motif in Trihelix transcription factor genes. (DOCX 13 kb)

Additional file 4: **Figure S2**. Trihelix family conservative domain feature analysis in foxtail millet. Stack height in different sites of amino acid shows conservative domains, the stack height of a single amino acid shows the relative frequency of the amino acid in this location. Red triangle shows that conservative core amino acids Trp (W) - 1, Trp (W) – 64 and Cys (C) -100. (JPG 740 kb)

Additional file 5: **Table S3**. RPKM values of millet Trihelix transcription factor genes in the different organs. (DOCX 17 kb)
