# Supplementary material for: Genomic, expressional, protein-protein interactional analysis of Trihelix transcription factor genes in Setaria italia and inference of their evolutionary trajectory
Source: BMC Genomics. 2018 Sep 12;19:665. doi: 10.1186/s12864-018-5051-9 (PMC6134603; doi:10.1186/s12864-018-5051-9)
Supplement: Supplementary file 1 — Table S1. Basic information of foxtail millet Trihelix transcription factor genes. (DOCX 15 kb) [file 12864_2018_5051_MOESM1_ESM.docx]

**Table S1** Basic information of foxtail millet Trihelix transcription factor genes.

| Gene  name | Genomic  location | Protein  size | Molecular weight | Isoelectric point |
| --- | --- | --- | --- | --- |
| Si1g016284.1 | Ch01:26657563..26665275 | 878 | 96360.6 | 8.7813 |
| Si1g016284.2 | Ch01:26657563..26665095 | 878 | 96360.6 | 8.7813 |
| Si1g016578.1 | Ch01:32877622..32880945 | 665 | 71649.4 | 5.596 |
| Si1g016578.2 | Ch01:32877622..32880945 | 590 | 63926.3 | 7.0511 |
| Si1g017397 | Ch01:26924335..26925764 | 402 | 45215.9 | 6.631 |
| Si1g017444 | Ch01:28081216..28082956 | 395 | 42286.6 | 8.4447 |
| Si1g017674 | Ch01:24910814..24914466 | 348 | 37409.5 | 4.9184 |
| Si1g019071 | Ch01:23487974..23489083 | 369 | 42499.2 | 6.1313 |
| Si1g019502 | Ch01:23469795..23470902 | 354 | 40618.1 | 6.8428 |
| Si2g030145 | Ch02:1024159..1026512 | 383 | 43320.2 | 7.299 |
| Si2g030430.1 | Ch02:39787191..39790934 | 337 | 36217.8 | 8.4452 |
| Si2g030430.2 | Ch02:39787191..39789256 | 335 | 36073.7 | 8.4452 |
| Si2g033157 | Ch02:6805665..6806410 | 246 | 27318.1 | 9.969 |
| Si3g022565 | Ch03:10705460..10709440 | 343 | 37074.9 | 10.5573 |
| Si5g002238 | Ch05:32899783..32903796 | 324 | 35141.5 | 10.2725 |
| Si5g004811 | Ch05:45440115..45441291 | 287 | 30946.8 | 9.2362 |
| Si6g014062 | Ch06:31255725..31257496 | 331 | 36722.7 | 5.1402 |
| Si7g009787.1 | Ch07:19211762..19215968 | 536 | 57346.5 | 6.9941 |
| Si7g009787.2 | Ch07:19211762..19215968 | 519 | 55124.8 | 7.2598 |
| Si7g009787.3 | Ch07:19211762..19216299 | 497 | 52943.2 | 7.5206 |
| Si7g009787.4 | Ch07:19211762..19215968 | 473 | 50130.4 | 8.6532 |
| Si7g009787.5 | Ch07:19211762..19215968 | 437 | 46298.7 | 8.2838 |
| Si7g010102.1 | Ch07:33614408..33617642 | 446 | 51032.7 | 6.6787 |
| Si7g010102.2 | Ch07:33615877..33618809 | 446 | 51032.7 | 6.6787 |
| Si7g010147 | Ch07:23301472..23305113 | 435 | 47414.9 | 7.9716 |
| Si7g010246 | Ch07:21189712..21191308 | 405 | 42744.4 | 6.583 |
| Si7g010590 | Ch07:18378501..18381361 | 327 | 35383.3 | 9.2012 |
| Si7g010820 | Ch07:29082816..29085865 | 273 | 31854.6 | 8.0882 |
| Si7g012121 | Ch07:26168663..26171924 | 675 | 72180 | 6.3201 |
| Si8g026391 | Ch08:3372080..3374128 | 434 | 49674.6 | 6.5811 |
| Si9g034382.1 | Ch09:58193087..58196848 | 753 | 79376.2 | 6.5764 |
| Si9g034382.2 | Ch09:58193087..58196848 | 752 | 79248 | 6.5764 |
| Si9g036121 | Ch09:50873611..50877505 | 385 | 42293 | 6.7964 |
| Si9g036682 | Ch09:36636641..36638111 | 317 | 33746.1 | 9.9519 |
| Si9g037484 | Ch09:49105056..49105906 | 212 | 23453.7 | 11.2729 |
| Si9g040176 | Ch09:9532729..9533944 | 368 | 39526.6 | 7.6825 |
