# Supplementary figures and images for: Genomic, expressional, protein-protein interactional analysis of Trihelix transcription factor genes in Setaria italia and inference of their evolutionary trajectory
Source: BMC Genomics. 2018 Sep 12;19:665. doi: 10.1186/s12864-018-5051-9 (PMC6134603; doi:10.1186/s12864-018-5051-9)

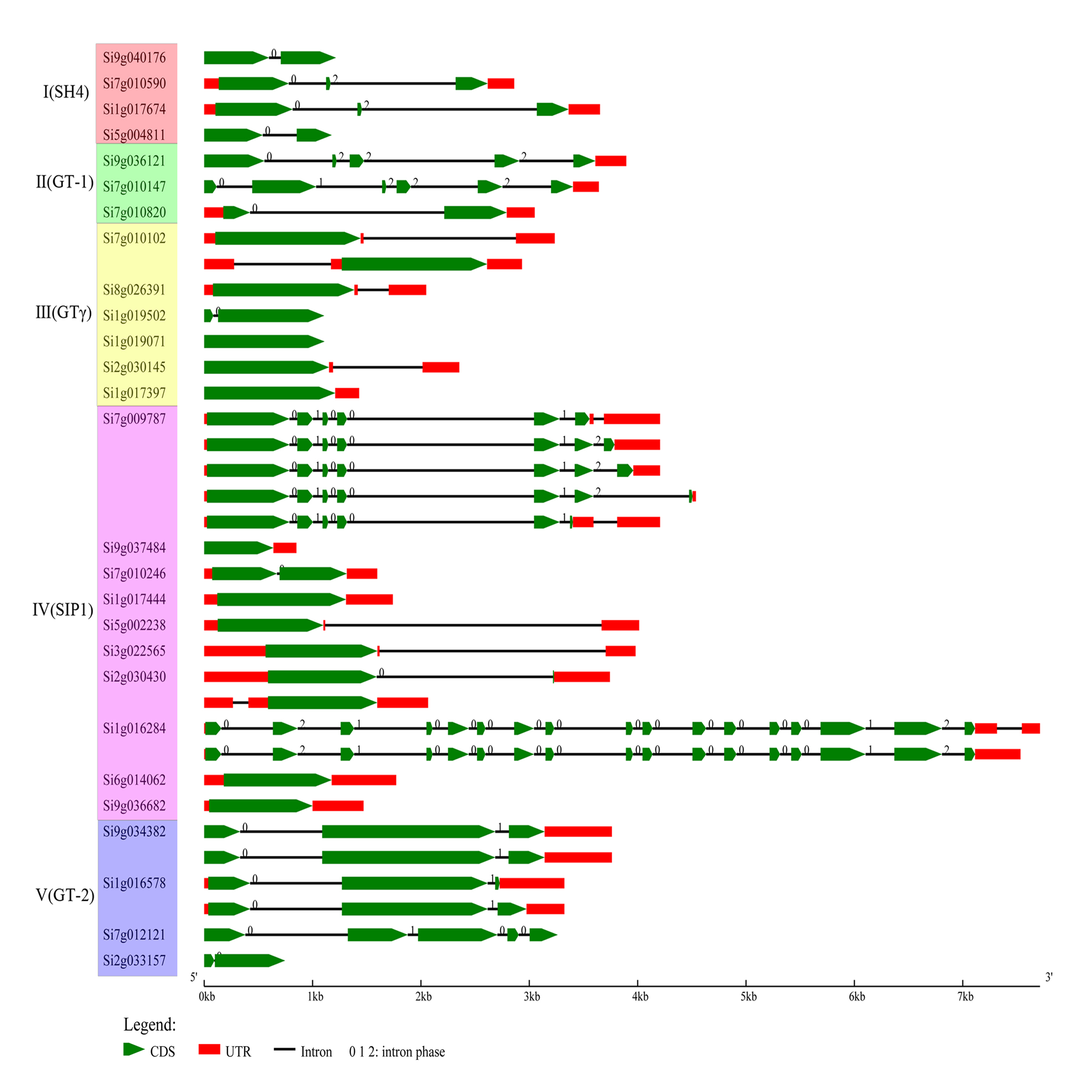

Supplement: Supplementary file 2 — Figure S1. Millet Trihelix family genetic structure analysis. (JPG 1489 kb) [file 12864_2018_5051_MOESM2_ESM.jpg]

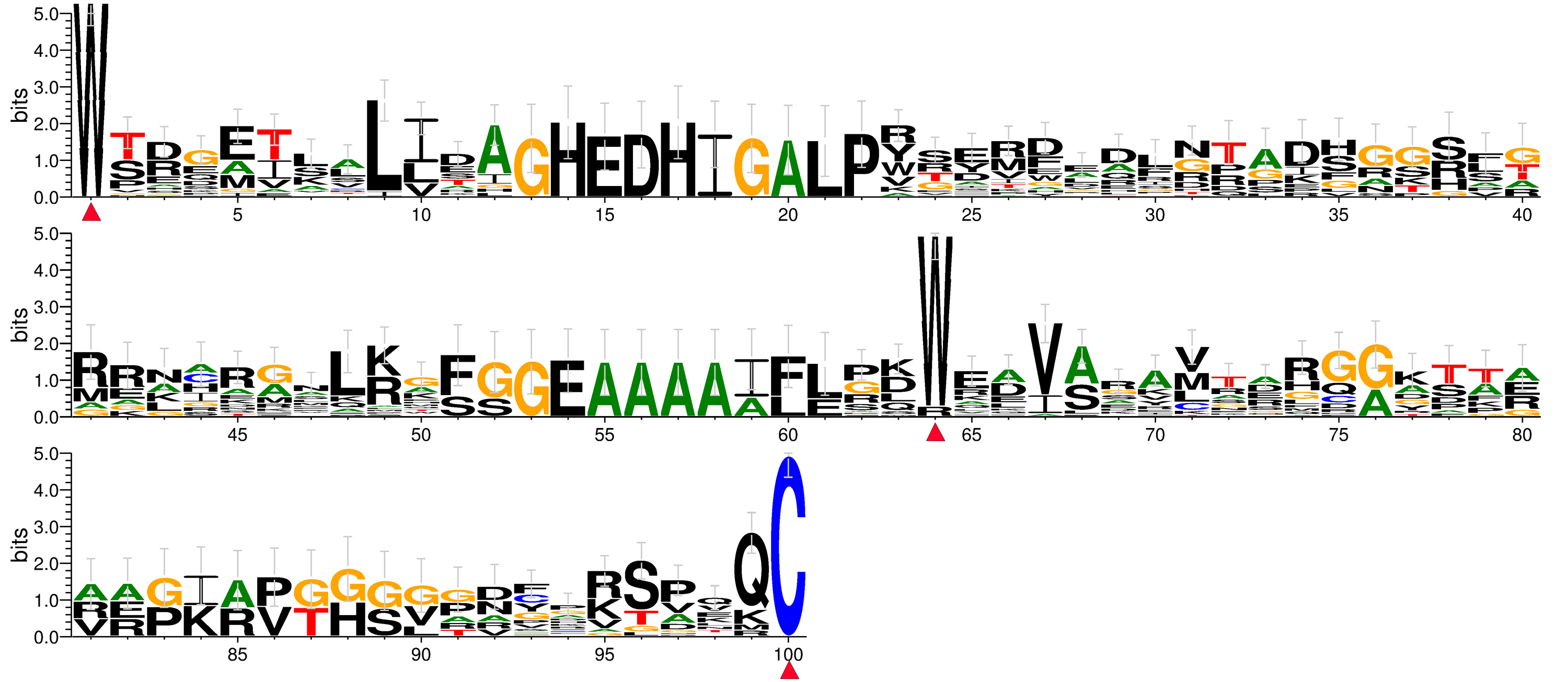

Supplement: Supplementary file 4 — Figure S2. Trihelix family conservative domain feature analysis in foxtail millet. Stack height in different sites of amino acid shows conservative domains, the stack height of a single amino acid shows the relative frequency of the amino acid in this location. Red triangle shows that conservative core amino acids Trp (W) - 1, Trp (W) – 64 and Cys (C) -100. (JPG 740 kb) [file 12864_2018_5051_MOESM4_ESM.jpg]
