# Supplementary material for: Genomic, expressional, protein-protein interactional analysis of Trihelix transcription factor genes in Setaria italia and inference of their evolutionary trajectory
Source: BMC Genomics. 2018 Sep 12;19:665. doi: 10.1186/s12864-018-5051-9 (PMC6134603; doi:10.1186/s12864-018-5051-9)
Supplement: Supplementary file 3 — Table S2. Conservative motif in Trihelix transcription factor genes. (DOCX 13 kb) [file 12864_2018_5051_MOESM3_ESM.docx]

**Table S2** Conservative motif in Trihelix transcription factor genes.

| Serial number | Amino acid number | Motif  sequence |
| --- | --- | --- |
| 1 | 200 | QCKNKIDNLKKRYKVECQRLASSSGGAVSHWPWFKKMEQIVGNSASPASSKPLATAEDEKPSQQQQQQHGSKRYPLSSAGPITVVGSSRVNPLSNPRWKRVLLKIGGTALAGPAPQNVDPKIIMLIAREVQVACHHGVEVAIVVGGRNIFCGDNWVAATGTDRASTYPIGMMASVMNSVLLQASLEKIGVETRVQTALMI |
| 2 | 70 | WPKEETHCLIQIRTEMDAHFQDAGLKGPLWEDISRKMRRLGYHRSAKKCKEKWENINKYYKKTKEHNKKR |
| 3 | 159 | CDDDFGLLGDDAHQPAAPPPQAPTTAQQPAPPPQPAQAFCFADAAVAPGAGAGAGSFAQVPEESNHHAERGKAAHHAKRTRERADEFSSDGGEYCSYINSGGSGGGGKKGRGGGSSGASDYRKDREEWTDGAISSLLDAYTDRFEQLNRGNLRGRDWED |
| 4 | 73 | TSGGRRNCTMMQKKGKWKLISKVMTERGCHVSPQQCEDKFNDLNKRYKRLTEILGRGTACRIVEKPELLEQMN |
| 5 | 44 | LSDKLKDEARKHLSSKHLHYEEMCSYHNHNRFCLPDDPALQRSL |
| 6 | 41 | LELAQSHLKWMRFSKEKDRELEKMRLENEMMKIENEQLELE |
| 7 | 51 | QEVAEPYVRRRAIRHLEKGRVVIFGGIGAGIGNPLFTTDTAAALRASEISM |
| 8 | 23 | RTPVQCKNRWDNLKKKYKKERAW |
| 9 | 41 | KEDCWSEGETVTLIDAWGERWVKLNRGNLRWPQWQEVADAV |
| 10 | 21 | TWPYFHQLDALYKCTHFCSGH |
| 11 | 57 | MMRFFEGMMKQVTEKQDEMQQVFIETIERWEQDRTIREEAWRRQEVARMNHEQDQLA |
| 12 | 74 | DRYIIIDDGIIFPDYDIFGPQKIHPDTTFIKKWKFKITTVCIWHGHGDHIGALYWVIHALDSTCPIFCPLFHME |
| 13 | 159 | IDDRLEDDRHPLPLTAADAVATNGVNQWNWRDTSTNGGDNQGTFGGRVIFVKWGDYTKRIGIDGTAEAIKEAIKSAFGLRTRRAFWLEDEDEVVRTLDRDMPIGTYTLHLDDGVTIKLCDANRMQTPEDKTFYTEDDFRDFLARRGWTLLREYGGYRNV |
| 14 | 200 | LIKKRLKEFGIFLSSRLKVLRIKKRFQAGPFEVEPIRVTHSVPDCCGLVLRCGDGIIFHTGDWKIDESPVDGKIFDREALEELSKEGVTLMMSDSTNVLSPGRSISESVVAGSLLRHISEAKGRVITTQFASNIHRIGSVKAAADLTGRKMVFVGMSLRTYLEAAFKDGKAPLDPSTLVKVEDMDAYAPKDLLVVTTGSQ |
| 15 | 51 | KGIVGDEEYGCPPRSNNNAPFEHISFREFAARGFSRMDMTAVTCCEENNIP |
| 16 | 21 | WHRMKWTDDMVKLLISAVSYI |
| 17 | 41 | TNYGSLDLHHNHMQFHDPNEGNQGFNNSQMPYNFPFHRNQQ |
| 18 | 57 | DLAQAIERFGEMYERVEAAKQKHAFEMERQRIDFLKELEVQRMQFFVNMQMELTRAK |
| 19 | 158 | RMVAEILRKMVRKYSGKRPDVIAVATENTTAGFSEHLEAKSSGNFGPSSATSHLSRSPARSLEGSYKTHPDNPDVEAEETLPEAVSTTPDDATTSSNGEAFFSSDLHQPKTLEHFWESFKSPTAVKIARIVNGGNKQNLGKIGILGKDPTQSAPAPVK |
| 20 | 39 | LPPPPLTPTPVPMAMPITPPPMQQQPPGIHPSPHPEHPP |
| 21 | 45 | LPVHGEMLYMKDHEDVGFGNGIRHHTCIKNGDMLGITHDINKVFP |
| 22 | 21 | HHHQQVHGTKKLKHDHEHGHC |
| 23 | 57 | YSDKMNIGNNTRVMKMMNKVTDLVPKIIMGKDSGLHVSGHRYKDENEDVLEIDKPQH |
| 24 | 29 | ERAMAASRDAAIINFLQRITGQTIQMPPV |
| 25 | 15 | VTDGQGKTTGGKSVE |
