# Supplementary material for: Genomic, expressional, protein-protein interactional analysis of Trihelix transcription factor genes in Setaria italia and inference of their evolutionary trajectory
Source: BMC Genomics. 2018 Sep 12;19:665. doi: 10.1186/s12864-018-5051-9 (PMC6134603; doi:10.1186/s12864-018-5051-9)
Supplement: Supplementary file 5 — Table S3. RPKM values of millet Trihelix transcription factor genes in the different organs. (DOCX 17 kb) [file 12864_2018_5051_MOESM5_ESM.docx]

| Original GeneID | GeneID | Root | Stem | Leaf | Spica |
| --- | --- | --- | --- | --- | --- |
| Millet_GLEAN_10012331 | Si9g040176 | 0.0975 | 0 | 0 | 1.748 |
| Millet_GLEAN_10030057 | Si7g010590 | 7.733 | 13.446 | 8.811 | 12.431 |
| Millet_GLEAN_10034757 | Si1g017674 | 9.562 | 11.895 | 15.661 | 10.642 |
| Millet_GLEAN_10036418 | Si5g004811 | 0 | 0.182 | 0.079 | 0.145 |
| Millet_GLEAN_10037920 | Si9g036121 | 8.832 | 14.887 | 11.094 | 13.638 |
| Millet_GLEAN_10029636 | Si7g010147 | 30.44 | 33.492 | 4.52 | 30.617 |
| Millet_GLEAN_10037280 | Si7g010820 | 6.021 | 10.023 | 7.495 | 11.079 |
| Millet_GLEAN_10003387 | Si7g010102 | 21.982 | 36.982 | 19.16 | 41.841 |
| Millet_GLEAN_10017094 | Si8g026391 | 4.8898 | 5.55 | 0.096 | 8.889 |
| Millet_GLEAN_10013824 | Si1g019502 | 0 | 0 | 0 | 0 |
| Millet_GLEAN_10013824 | Si1g019071 | 0 | 0 | 0 | 0 |
| Millet_GLEAN_10003288 | Si2g030145 | 0.264 | 2.012 | 0.439 | 4.835 |
| Millet_GLEAN_10023807 | Si1g017397 | 20.797 | 33.391 | 7.066 | 42.277 |
| Millet_GLEAN_10029973 | Si7g009787 | 33.555 | 26.721 | 16.013 | 35.507 |
| Millet_GLEAN_10013555 | Si9g037484 | 0.256 | 0.366 | 0.267 | 0.29 |
| Millet_GLEAN_10033811 | Si7g010246 | 15.342 | 8.903 | 3.668 | 17.571 |
| Millet_GLEAN_10035846 | Si1g017444 | 9.71 | 6.533 | 5.217 | 11.784 |
| Millet_GLEAN_10025305 | Si5g002238 | 37.873 | 24.519 | 4.783 | 19.588 |
| Millet_GLEAN_10028836 | Si3g022565 | 51.973 | 36.31 | 10.609 | 48.526 |
| Millet_GLEAN_10027816 | Si2g030430 | 63.459 | 43.092 | 19.535 | 37.803 |
| Millet_GLEAN_10023822 | Si1g016284 | 9.835 | 26.516 | 6.959 | 31.753 |
| Millet_GLEAN_10022662 | Si6g014062 | 19.491 | 21.038 | 10.515 | 28.86 |
| Millet_GLEAN_10008612 | Si9g036682 | 96.682 | 43.734 | 13.068 | 42.38 |
| Millet_GLEAN_10005068 | Si9g034382 | 1.456 | 9.215 | 0.869 | 15.46 |
| Millet_GLEAN_10002835 | Si1g016578 | 3.321 | 10.924 | 0.211 | 36.274 |
| Millet_GLEAN_10021092 | Si7g012121 | 5.135 | 50.947 | 0.856 | 65.305 |
| Millet_GLEAN_10017401 | Si2g033157 | 0.359 | 1.709 | 1.306 | 26.936 |

**Table S3** RPKM values of millet Trihelix transcription factor genes in the different organs.
